# The Effect of *Mentha Pulegium* on Healing of Burn Wound Injuries in Rat

**DOI:** 10.29252/wjps.8.1.43.

**Published:** 2019-01

**Authors:** Reza Vaghardoost, Yaser Ghavami, Behnam Sobouti

**Affiliations:** 1 Department of Aesthetic and Reconstructive Surgery, Shahid Motahari Burn Hospital, Iran University of Medical Sciences, Tehran, Iran;; 2 Burn Research Center, Shahid Motahari Hospital, Iran University of Medical Sciences, Tehran, Iran;; 3 Department of Pediatric Infectious Diseases, Burn Research Center, Shahid Motahari Burns Hospital, Iran University of Medical Sciences, Tehran, Iran

**Keywords:** *Mentha pulegium*, Burn, Wound, Healing, Rat

## Abstract

**BACKGROUND:**

Burn is one of the most common injuries and the 4th common cause of trauma globally. This study was designed to evaluate the effect of the *Mentha pulegium* extract on second degree burn injuries in rats assessing histopathologic and macroscopic.

**METHODS:**

*M. Pulegium* extract dressings was used as a treatment to deep dermal contact burns in rats, compared with two control groups of vaseline dressing and simple dressing by normal saline. After creating second-degree burn on the dorsum of rats, the treatments were applied for 15 min in three groups. Wound dressings were performed on days 1, 3, 7, 14 and 21^st^ and at the same time macroscopic assessment was performed using a digital camera and software processing of photos. Pathologic evaluation of skin specimens was undertaken on days 1, 3,7, 14 and 21^st^. Wound healing parameters such as epithelialization, angiogenesis, granulation tissue formation, inflammatory cells were compared between these 3 groups.

**RESULTS:**

Application of *M. Pulegium* extract on second degree burn wounds significantly decreased burn surface area and increased fibroblasts in comparison to simple dressing. There was not any statistically significant relationship between *M. Pulegium* extract treated group and vaseline treated or simple dressing groups on other wound healing parameters.

**CONCLUSION:**

This study delineated that *M. Pulegium* extract had a positive effect on healing process of second degree burns.

## INTRODUCTION

Burn is one of the most common injuries and the 4^th^ common cause of trauma globally.^1^ Burn injuries can lead to serious morbidities such as infections, scars and disabilities; as well as psychosocial and economical problems.^2^ In a study in United States, approximately 700000 individuals referred to the emergency wards seeking treatment for burn injuries and amongst them, 45000 injured patients needed hospitalization.^3^^,^^4^ In Iran, the mortality rate was shown to vary from 1.4 to 9.7 per one hundred thousand.^5^

Management and treatment of burn injuries are various and numerous in different countries and cultures.^6^ However, the mainstay of therapy is to resuscitate patients with fluid and prevent the infections of the burn wounds until the body regenerates the damaged tissues and skin. During the years, specific herbals have been developed and used to facilitate and accelerate the process of healing and tissue regeneration. There are some studies that explain alternative therapies in burn management.^6^^-^^9^ For example moist exposed burn ointment which is an herbal remedy for burn treatment was developed and used in China.^10^^,^^11^ Saffron (*Crocus sativus*) extract was used for healing of second-degree burn wounds in rats.^12^ Green tea extract was effective for treatment of second degree burn wounds.^13^ There are other studies supporting the effect of herbal remedies in treatment of burn wounds.^14^^-^^20^ Traditionally, an extract from the leaves of a plant called *M. pulegium* has been used to alleviate first and second degree burn wounds in north rural areas of Iran. This study was conducted to evaluate the effect of *M. pulegium* extract on second degree burn injuries in rats.

## MATERIALS AND METHODS

*M. pulegium* extract was obtained by maceration method. Leaves of *M. pulegium* were converted to a powder by grinding. About 20 g of powder were mixed with 300 ml ethanol (70%) at room temperature for 48 h. Then, the mixture was filtered from paper-making machine to extract the liquid. After preparing the liquid extract, it was placed in 10 plates in a water bath for 48 h at 70°C temperature until it was completely dried. Then, 2 g of alcoholic extract of *M. pulegium* was mixed with 100 ml saline; and finally 2% of the alcoholic extract of *M. pulegium* was obtained. This extract was standardized.

In this experimental study, 30 Wistar albino rats weighing approximately 300-350 g were used. All the rats were healthy and they were screened for animal diseases by a veterinarian. The rats were kept in separate shelves in the animal laboratory with 12 h light-dark cycle and temperature of 22°C *ad libitum*. All animals were housed in sterilized containers. They were randomly divided into three groups (*M. pulegium* extract, vaseline, and simple dressing) with 10 rats in each. To create a burn wound in animals, the rats were anesthetized by intramuscular injection of ketamine (60 mg/kg) and xylazine (10 mg/kg). After shaving their backs hair by a blade, a deep second degree burn wound was created by a metal cube with dimensions of 2×3×1 cm that was heated to 105°C for 15 seconds and an area of about 6 cm^2^ was burned. Thereafter, treatment was initiated for these three groups with different materials.

The animals were resuscitated with an intraperitoneal injection of 5 ml normal saline solution. Dressings were performed on days 1, 3, 7, and 14^th^. The rats were given anesthesia while taking photos and also during dressing change. Each test animal was held in a good position and wound margin was traced on a transparent plastic sheet using a fine-tipped pen. The wounds were measured by a ruler on each rat and recorded. Thereafter, photographs of burned areas were taken using a digital camera (Canon power-shot D10) on days 1, 3, 7, 14 and 21^st^. Sizes and areas of burn surface were measured by a standard metric and displayed as cm^2^ using the software Image J on each experiment day. 

The area of the wounds on the first day was considered as 100% and wound areas on subsequent days were compared with the wound area on the initial days. Re-epithelialization was evaluated on days 1, 3, 7, 14 and 21^st^ (the last day of treatment period). For this purpose, skin tissue samples were taken for histological studies with a small excision containing part of the wound area. Tissues were fixed in 10% formalin. Paraffin-embedded sections (5-μm thick) were prepared and stained with hematoxylin and eosin. Light microscopy was used to evaluate the pathological changes, e.g., granulation tissue formation and re-epithelialization in wounds and their comparison with the normal tissue. 

The severity of inflammation in treated areas was evaluated by counting the number of inflammatory cells (neutrophils, lymphocytes, and fibroblasts) in 4 different fields (x100) using an ocular micrometer and the mean number of these 4 fields was recorded. The number of blood vessels was counted in 4 different fields (x400) and the mean number was recorded (Table 1). All data were analyzed using statistical software SPSS (version 18; Chicago, Illinois, USA). One-way ANOVA test was used to compare means. A *p* value less than 0.05 was considered as significant. This study was confirmed by the Ethic Committee of Iran University of Medical Sciences, Tehran, Iran.

**Table 1:** Comparison of parameters indicating healing of the burn wound in 3 different groups on days 1, 3, 7, 14 and 21

**Parameters**	**Days**	# **Case group (treated with MPE)**	# **Vaseline treated group**	# **Simple dressing (treated with normal saline)**	* **P value** ^1^	** **P value** ^ 2^
Neutrophils	1	14±3.2	15±4.1	14±2.6	0.32	0.93
	3	12±2.4	14±2.3	12±1.4	0.46	0.85
	7	11±2.2	12±3.1	12±2.6	0.43	0.37
	14	10±3.6	11±2.2	11±3.7	0.52	0.61
	21	8±2.2	10±3.4	11±2.8	0.63	0.32
Lymphocytes	1	9±3.1	9±3.4	9±3.5	0.59	0.53
	3	10±2.5	9±2.2	8±3.1	0.23	0.09
	7	12±3.1	10±2.6	10±2.1	0.16	0.22
	14	17±3.2	14±2.1	12±2.3	0.28	0.08
	21	32±2.4	25±1.5	21±2.6	0.12	0.07
Fibroblasts	1	6±2.6	5±2.2	4±2.3	0.43	0.21
	3	14±3.3	12±2.4	12±2.1	0.51	0.62
	7	19±2.5	14±3.2	10±2.5	0.39	0.18
	14	25±3.2	22±2.1	19±3.1	0.08	0.05
	21	34±2.8	28±3.5	23±3.6	0.07	0.04
Angiogenesis	1	2±1.1	1±0.05	1±0.2	0.62	0.74
	3	4±1.8	3±1.5	3±2.4	0.53	0.24
	7	7±2.2	6±2.1	5±2.8	0.65	0.41
	14	7±1.5	6±2.7	6±2.1	0.73	0.62
	21	10±3.2	8±2.3	8±3.3	0.09	0.15
Granulation tissue	1	1±0.15	1±0.05	1±0.03	0.92	0.86
	3	2.1±0.25	1.4±0.14	1.5±0.22	0.44	0.83
	7	1.2±0.45	1.1±0.12	1±0.02	0.72	0.66
	14	0.8±0.15	0.5±0.32	0.3±0.02	0.36	0.08
	21	0.5±0.20	0.4±0.15	0.2±0.35	0.67	0.34

# All values are depicted in Means±SD.

* The statistical difference between MPE treated group and Vaseline treated group.

** The statistical difference between MPE treated group and Simple dressing (normal saline) group.

1,2 P value less than 0.05 is considered statistically significant.

## RESULTS

**Fig. 1:**
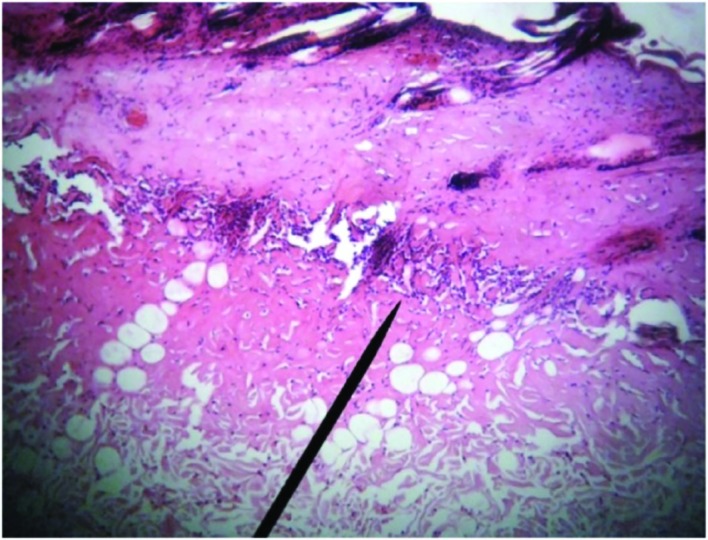
Microscopic view of burned area in day 1^st^ post-burn. Increased necrosis area, inflammatory cells accumulation above necrotic area and fibrin clot are visible (H&E, ×400)

Histological findings indicated a progressive improvement in wound healing during 21 days after starting treatment. The slides from *M. pulegium* extract treated wounds showed significant wound healing with completed re-epithelialization of epidermis. On day 1^st^ post-burn, epidermis became necrotic due to coagulation. Necrotic basal layer cells were seen arranged in column-fencing formation. Edema was present between epidermis and dermis. Collagenous fibers in the superficial layer of dermis were denatured, swollen and loosely arranged while epithelia of the dermal adnexal epithelia were also denatured. Contracted nucleoli of capillary endothelium, blood clot or stasis in lumen with little infiltration of neutrophil were observed. Only collagenous fibers and dermal appendages in deep layer of dermis were approximately normal (Figure 1).

**Fig. 2:**
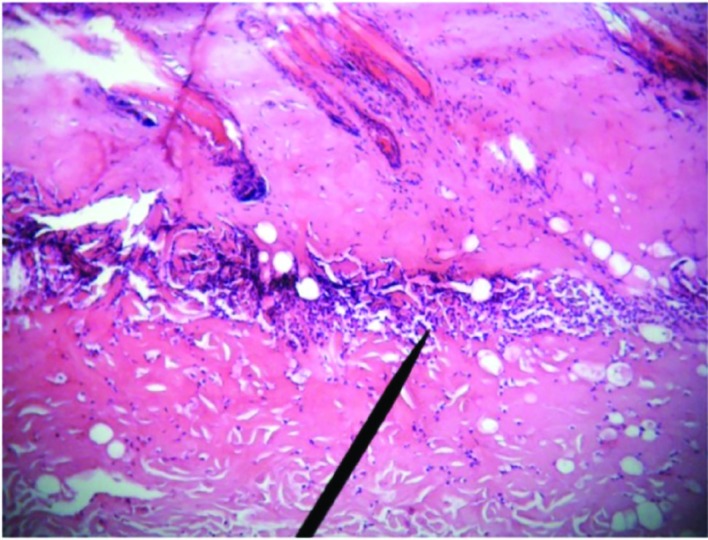
Microscopic view of burned area in case group on 3^rd^ day after treatment which depicts full thickness skin necrosis and accumulation of inflammatory cells (H&E, ×100)

On day 3 post-burn, necrotic epidermal cells progressed to vacuolation with mild edema between epidermal and dermal layers. Collagenous fibers in the superficial dermis had hyaline degeneration in deep tissue, structure was loose, and blood vessels were slightly swollen with congestion. Scattered inflammatory cells (mainly neutrophils) infiltrated the tissue (Figure 2). 

**Fig. 3:**
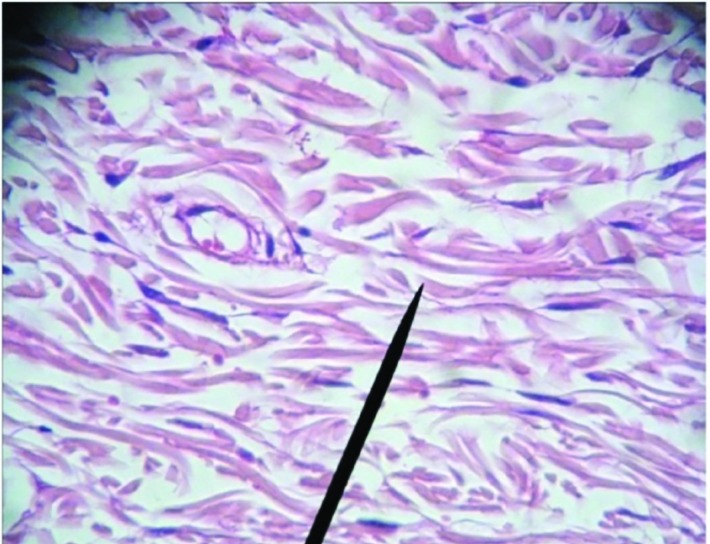
Microscopic view of burned area in case group on 7^th^ day after treatment which depicts granulation tissue formation, collagen fibrils and fibroblasts (H&E, ×400)

**Fig. 4:**
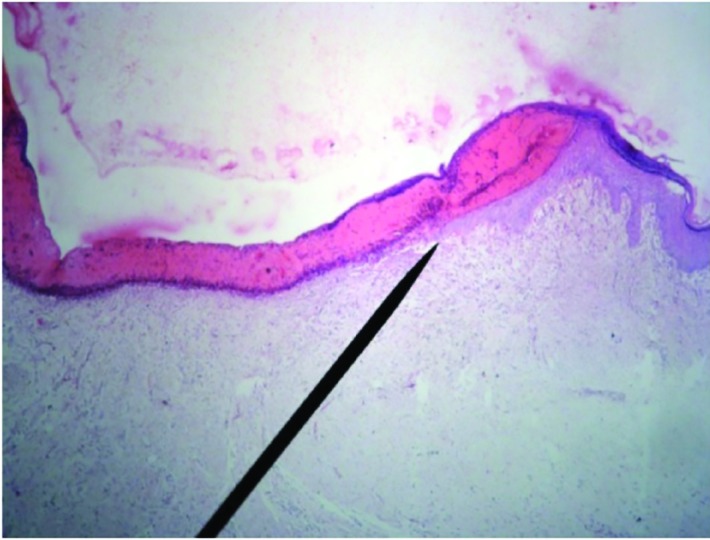
Microscopic view of burned area in case group at 14^th^ day after treatment which depicts newly formed epithelium (H&E, ×40)

On day 7^th^ when necrotic epidermis exfoliated, superficial tissues in the dermis liquefied and loosened due to necrosis and neutrophilic infiltration (occasional lymphocyte and mononuclear-macrophages) and granulation tissue formation (Figure 3). On day 14^th^ post-injury, an inflammatory exudation layer replaced the necrotic layer whose liquefaction was now accomplished. The underlying residual adnexal epithelia, fibroblasts and endothelia showed active proliferation. The new epithelium layer was seen (Figure 4). 

**Fig. 5:**
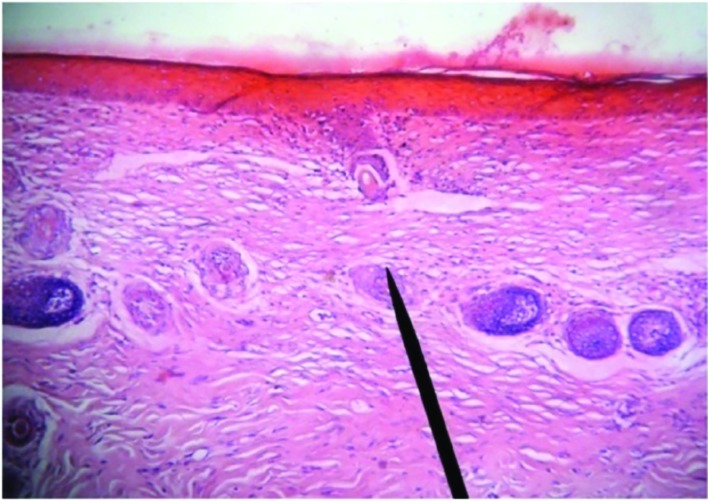
Microscopic view of burned area in case group at day 21^st^ after treatment which depicts completely formed epithelium (H&E, ×100)

On day 21^st^ post-burn, regenerated “epithelial islands” grew vertically, then migrated toward and covered the wounds. Neo-formed capillaries were noticed and granulation tissues were formed. The infiltration of inflammatory cells (mainly lymphocytes) in the dermis was still present, especially in the periphery of the regenerated skin appendages. After healing, regenerated skin appeared to be almost normal in structure and majority of skin appendages were restored completely. Infiltration of certain inflammatory cells and few macrophages in dermis persisted (Figure 5).

**Fig. 6:**
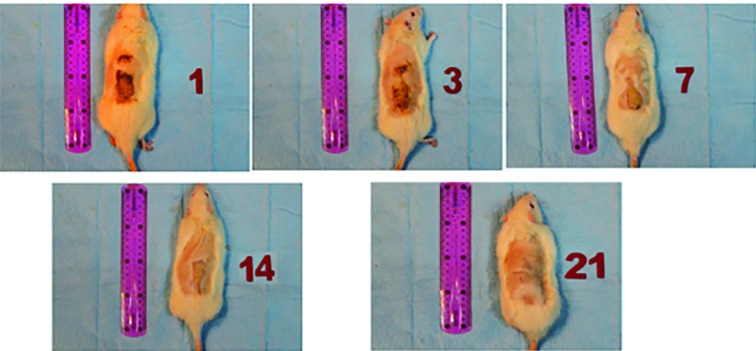
Progressive improvement of burn wound in rats treated with *Mentha pulegium* extract

Comparison of parameters indicating healing of the burn wound (angiogenesis, granulation formation, inflammatory cells and fibroblasts) in these 3 different groups on days 1,3, 7, 14 and 21^st^ were shown in Table 1. Burned areas were evaluated and compared on days 1, 3, 7, 14 and 21^st^. The evident difference of healing process was observed after day 14^th^ (Table 2). Average burned area was reduced significantly in the group who was treated with *M. pulegium* extract. The average burned area was statistically significant between *M. pulegium* extract treated group and simple dressing group (*p*=0.03); however, there was no statistically significant difference between *M. pulegium* extract and vaseline treated groups. Improvement of burn wounds using *M. pulegium* extract was shown in Figure 6.

**Table 2:** Comparison of Burned Surface Areas (BSA) in 3 groups in days 1, 3, 7, 14 and 21

**Burned Surface Area (BSA)**	***Mentha pulegium***	**Vaseline ointment**	**Simple dressing**	*P value ^1^	**P value ^ 2^
BSA (cm^2^) day 1				0.72	0.84
Mean N Std Min Max	5.72 10 2.25 4.41 7.29	5.22 10 1.74 4.29 7.53	5.41 10 2.31 4.25 7.41		
				0.56	0.63
BSA (cm^2^) day 3 Mean N Std. Min Max	4.26 10 1.26 4.37 5.52	5.11 10 1.93 4.11 7.21	5.22 10 1.62 4.15 7.25		
				0.27	0.34
BSA (cm^2^) day 7 Mean N Std. Min Max	3.21 10 1.58 2.91 4.83	4.67 10 1.33 2.89 5.66	4.87 10 1.17 5.73 5.96		
				0.11	0.08
BSA (cm^2^) day 14 Mean N Std. Min Max	2.35 10 1.82 .38 3.29	3.38 10 1.23 2.50 4.78	4.27 10 1.53 2.84 5.36		
BSA (cm^2^) day 21				0.09	0.03
Mean N Std. Min Max	1.12 10 0.42 .00 1.82	2.63 10 1.83 2.50 3.63	3.78 10 1.64 2.84 4.41		

## DISCUSSION

The study results indicated a staged pathological change of burn wound when treated with *M. pulegium* extract. Burn area decreased significantly in *M. pulegium* extract treated group. Our results showed that topical application of *M. pulegium* extract was efficacious in treatment of second degree burn wounds in comparison to vaseline and simple (normal saline) dressings. At the end of the study (day 21^st^), microscopic and macroscopic findings indicated that treatment of deep second-degree burn wounds with *M. pulegium* extract results in physiological regeneration with minimal scar formation. 

*M. pulegium* commonly known as pennyroyal belongs to the *Mentha* species is a native species found in Europe, North Africa and in Asia Minor and near East.^19^ Previous studies have shown that *M. pulegium* has antioxidative, antimicrobial, spasmolytic and relaxant activities.^20^^-^^23^ The effect of *M. pulegium* on improvement of the burn lesion can be attributed to any of the aforementioned effects or all of them together. The relaxant and spasmolytic effects of *M. pulegium* have been ascribed to the “Pulegone” which possibly relaxes the smooth muscles via increasing the participation of extracellular calcium. Stimulation of calcium sensitive potassium channels hyperpolarize the cell and causes relaxation. The relaxation of the smooth muscles in the vessels’ walls will increase the blood perfusion to the injured tissue. Antimicrobial effect of the *M. pulegium* has been studied in vitro using its oil extract.^24^

The antimicrobial activity of *M. pulegium* has been attributed to *Piperitone* and *Piperitenone *which have activity against Gram-positive bacteria especially *Staphylococcus aureus*.^23^ It has also been shown that piperitone completely inhibited *Aspergillus flavus*.^25^ *M. pulegium* has been shown to be effective as a protoscolicidal agent too and it killed *Echinococcus granulosus* proroscoleces.^26^ The antioxidant activity of *M. pulegium* also helps to facilitate and accelerate the improvement of burn tissue by increasing the activity of superoxide dismutase and glutation peroxidase.^27^

There is another study on naturally-derived products in treatment of burn wounds which showed healing effect of biomaterials in first and second degree burn wounds. For example, in a case-control study by Gupta and colleagues, honey dressing sterilized the wounds in less time, enhanced healing and had a better result in treating hypertrophic scars and post-burn contractures in comparison to silver sulfadiazine.^6^ Scientists in China developed an alternative therapy for treating burn wounds as herbal remedy which was named moist exposed burn ointment. They claimed that it was ideal wound dressing option for burns.^11^

Thereafter, numerous studies have been conducted using moist exposed burn ointment for burn treatment. These studies claimed that moist exposed burn ointment can reduce wound healing time, bacterial colonization, and the need for analgesics or antibiotics and also it results in a better aesthetic outcome. Moist exposed burn ointment is an oil-based ointment composed of sesame oil and some other plant ingredients.^10^^,^^11^^,^^14^^-^^16^ However, in our study, the simple dressing which was a combination of a sterile gauze and normal saline did not show any significant improvement in burn wounds. Therefore, the moisture alone is not a factor for improvement of burn wounds. There should be an antioxidant and antimicrobial effects of moist exposed burn ointment that helps improving the burn wounds. It was clarified that anti-inflammatory and antimicrobial effects of moist exposed burn ointment was due to beta-sitosterol and berberine, respectively.^28^

Alternative medicine has been used in treating burn wounds since ancient times. Herbal products such as moist exposed burn ointment, *Aloe vera*, *Aloe littoralis*, *Aloe saponaria* haw and green tea have been effective in treatment of burn injuries in animal models.^10^^-^^18^^,^^28^^,^^29^ On the other hand, using stem cells or skin grafts in burn injuries is costly and limited for developing countries, therefore, alternative therapies for second degree burn wounds has become a reasonable modality for cure.^30^ Our results showed that *M. pulegium* extract was beneficial in treating second degree burn injuries, but the side effects of this product are not yet determined. Also we are intending to determine the main ingredients and substances in *M. pulegium* which may contribute to its wound healing effects and formulize a biomaterial for treatment of second degree burn wounds. 
